# Personalized Clinical Phenotyping through Systems Medicine and Artificial Intelligence

**DOI:** 10.3390/jpm11040265

**Published:** 2021-04-02

**Authors:** Alfredo Cesario, Marika D’Oria, Francesco Bove, Giuseppe Privitera, Ivo Boškoski, Daniela Pedicino, Luca Boldrini, Carmen Erra, Claudia Loreti, Giovanna Liuzzo, Filippo Crea, Alessandro Armuzzi, Antonio Gasbarrini, Paolo Calabresi, Luca Padua, Guido Costamagna, Massimo Antonelli, Vincenzo Valentini, Charles Auffray, Giovanni Scambia

**Affiliations:** 1 Open Innovation Unit, Scientific Directorate, Fondazione Policlinico Universitario A. Gemelli IRCCS, 00168 Rome, Italy; alfredo.cesario@policlinicogemelli.it; 2 Neurology Unit, Fondazione Policlinico Universitario A. Gemelli IRCCS, 00168 Rome, Italy; francescobove86@gmail.com (F.B.); paolo.calabresi@policlinicogemelli.it (P.C.); 3 Department of Neurosciences, Università Cattolica del Sacro Cuore, 00168 Rome, Italy; 4 CEMAD—IBD Unit—Internal Medicine and Gastroenterology Unit, Fondazione Policlinico Universitario A. Gemelli IRCCS, 00168 Rome, Italy; gpp.privitera@icloud.com (G.P.); alessandro.armuzzi@policlinicogemelli.it (A.A.); antonio.gasbarrini@policlinicogemelli.it (A.G.); 5 Department of Medicine and Translational Surgery, Università Cattolica del Sacro Cuore, 00168 Rome, Italy; 6 Surgical Endoscopy Unit, Fondazione Policlinico Universitario A. Gemelli IRCCS, 00168 Rome, Italy; ivo.boskoski@policlinicogemelli.it (I.B.); guido.costamagna@policlinicogemelli.it (G.C.); 7 Cardiology Unit, Fondazione Policlinico Universitario A. Gemelli IRCCS, 00168 Rome, Italy; daniela.pedicino@policlinicogemelli.it (D.P.); giovanna.liuzzo@policlinicogemelli.it (G.L.); filippo.crea@policlinicogemelli.it (F.C.); 8 Radiation Oncology Unit, Fondazione Policlinico Universitario A. Gemelli IRCCS, 00168 Rome, Italy; luca.boldrini@policlinicogemelli.it (L.B.); vincenzo.valentini@policlinicogemelli.it (V.V.); 9 High Intensity Neurorehabilitation Unit, Fondazione Policlinico Universitario A. Gemelli IRCCS, 00168 Rome, Italy; carmen.erra@policlinicogemelli.it (C.E.); claudia.loreti@policlinicogemelli.it (C.L.); luca.padua@policlinicogemelli.it (L.P.); 10 Anesthesia, Resuscitation, Intensive Care and Clinical Toxicology Unit, Fondazione Policlinico Universitario A. Gemelli IRCCS, 00168 Rome, Italy; massimo.antonelli@policlinicogemelli.it; 11 European Institute for Systems Biology and Medicine (EISBM), 69390 Vourles, France; cauffray@eisbm.org; 12 Scientific Directorate, Fondazione Policlinico Universitario A. Gemelli IRCCS, 00168 Rome, Italy; giovanni.scambia@policlinicogemelli.it; 13 Gynecological Oncology Unit, Fondazione Policlinico Universitario A. Gemelli IRCCS, 00168 Rome, Italy

**Keywords:** personalized medicine, systems medicine, gastroenterology, digestive endoscopy, cardiology, neurology, neurorehabilitation, artificial intelligence, machine learning, P4 medicine

## Abstract

Personalized Medicine (PM) has shifted the traditional top-down approach to medicine based on the identification of single etiological factors to explain diseases, which was not suitable for explaining complex conditions. The concept of PM assumes several interpretations in the literature, with particular regards to Genetic and Genomic Medicine. Despite the fact that some disease-modifying genes affect disease expression and progression, many complex conditions cannot be understood through only this lens, especially when other lifestyle factors can play a crucial role (such as the environment, emotions, nutrition, etc.). Personalizing clinical phenotyping becomes a challenge when different pathophysiological mechanisms underlie the same manifestation. Brain disorders, cardiovascular and gastroenterological diseases can be paradigmatic examples. Experiences on the field of Fondazione Policlinico Gemelli in Rome (a research hospital recognized by the Italian Ministry of Health as national leader in “Personalized Medicine” and “Innovative Biomedical Technologies”) could help understanding which techniques and tools are the most performing to develop potential clinical phenotypes personalization. The connection between practical experiences and scientific literature highlights how this potential can be reached towards Systems Medicine using Artificial Intelligence tools.

## 1. Introduction

Biological systems are complex and thus characterized by emergent properties, meaning that they exert properties that cannot be explained by the function of the single components of the system, but only by their interactions [1]. In a similar fashion, human diseases follow non-linear dynamics, where small alterations can produce effects of significant and unexpected magnitude over time [2]. The traditional top-down approach to medicine, founded on population-based observations and clinicopathological classification of diseases, has performed egregiously as long as single etiologic factors were directly responsible for the advent human diseases (i.e., infectious diseases). However, this approach fails to significantly impact the natural history of the new prevalent diseases that affect western populations, which are chronic conditions with multifactorial etiology [3].

The understanding of complex disorders required a shift in the traditional research paradigm and the adoption of a systemic, integrative, and personalized approach to the patient. Every person is unique; hence, the goal of Personalized Medicine (PM) is to move from a “one-size-fits-all” approach to a more tailored one who takes into account the complexity of each patient. However, the concept of PM may assume different (and sometimes paradoxical) interpretations [4,5,6,7]. On one hand, it seems to coincide with the advent of omic sciences (such as genomics, proteomics, metabolomics) with particular regard to Genetic and Genomic Medicine [8]. By looking for a definition of the term “Personalized Medicine”, the MeSH Browser redirects the research to “Precision Medicine” defined as follows: “Clinical, therapeutic and diagnostic approaches to optimal disease management based on individual variations in a patient’s genetic profile” [9]. On the other hand, PM has to embrace the whole complexity of a person, and broaden its perspectives to non-strictly medical data, such as the environment, lifestyle, or individual choices [10,11].

Indeed, technical advances in recent decades have led to the generation of a huge amount of biological data starting from a small quantity of samples; omics techniques allow for producing very large and complex datasets measuring large numbers of analytes, whose interpretation requires sophisticated computational approaches to draw significant conclusions [12]. Only a small part (about 2%) of the human genome encodes protein, while the number of actual proteins produced is much higher, since a single gene might have several splicing variants, and once translated, proteins might be subjected to post-translational modification in response to different environmental conditions [13]. Furthermore, the non-coding genome has been recognized as a key determinant in the complexity of pathophysiological mechanisms since thousands of non-coding RNAs (ncRNAs) are involved in the regulation of both genes and proteins. Despite the fact that some disease-modifying genes affect disease expression and progression, many complex conditions cannot be understood only through a “reductive” approach [14,15], especially when other factors play an important role on health. Brain disorders, cardiovascular and gastroenterological diseases can be paradigmatic examples in demonstrating how different pathophysiological mechanisms can lead to the same clinical phenotype.

Besides the implementation of multiomic explorations of disease pathways, the endeavor of this article is to consider the systemic precepts of PM (in its wider definition of P4 Medicine [preventive, predictive, participative, and personalized]) [16,17,18] to achieve a more accurate personalization of clinical phenotyping in complex conditions. Far from being a collateral emergence, Artificial Intelligence (AI) solutions might help analyzing multiple clinical features and stratifying them by common biological properties to achieve deep phenotyping.

## 2. The Example of Brain Disorders

As populations are growing and ageing, neurological disorders are increasingly recognized as major causes of death and disability worldwide [19]. The clinical presentation of neurological disorders is heterogeneous, resulting from a complex interaction of multiple genetic and environmental factors. Thus, patients with the same disease may present different phenotypes with a broad range of signs and symptoms. Conversely, however, in some cases the same phenotype may be caused by different conditions. Emerging approaches and technologies in neurosciences are allowing for the personalization of medical care in diagnosis, prognosis, and treatment of several neurological disorders [20].

### 2.1. Degenerative Parkinsonisms

Parkinsonian syndrome typically includes bradykinesia, extrapyramidal rigidity, and rest tremor. It is a common finding in older people, recognizing several underlying pathological conditions often of a neurodegenerative nature [21]. Despite advances in neuroimaging and basic sciences, to date, in-vivo diagnosis of Parkinson’s Disease (PD) and other degenerative parkinsonisms remains primarily clinical. In these disorders, misdiagnosis is common, and the diagnosis can be changed in many patients after a follow-up of a few years [22]. In recent years, several biomarkers have been purposed in differential diagnosis of parkinsonian disorders (Table 1). These molecules, detected in biological samples [mainly blood and cerebrospinal fluid (CSF)], are indicators of normal biological or pathogenic processes [23].

Degenerative parkinsonisms are neuropathologically characterized by accumulation of proteinaceous material in and around neurons and glial cells. The proteins that make up these aggregates, α-synuclein, Aβ-40, Aβ-42 and phosphorylated tau (p-tau), have a central role in the pathogenesis of these conditions [29]. The dosage of these proteins in CSF may be helpful to distinguish different pathologies. In particular, protein misfolding cyclic amplification (PMCA), a technique used to detect α-synuclein aggregates, may discriminate between samples of CSF from patients with different α-synucleinopathies, as multiple system atrophy (MSA) versus PD and dementia with Lewy bodies (DLB), with an overall sensitivity higher than 95% [24,25]. Indeed, CSF total α-synuclein, Aβ-42 and p-tau/total tau ratio have been suggested in differential diagnosis of α-synucleinopathies, although with inconsistent results [23,26,27].

Another biomarker purposed is Neurofilament light chain (Nfl), an unspecific marker of axonal degeneration, which has high accuracy in differentiating PD from atypical parkinsonisms [MSA, progressive sopranuclear palsy (PSP) and cortico-basal degeneration (CBD)], but not in discriminating among different subtypes of atypical parkinsonisms [26]. In fact, increased blood and CSF levels of Nfl reflect a more aggressive neurodegenerative process, characteristic of atypical parkinsonisms more than PD.

Furthermore, another mechanism involved in pathophysiology of different neurodegenerative conditions is neuroinflammation, several neuroinflammatory biomarkers (acute phase proteins, cytokines and markers of microglial activation) have been studied in different conditions. Interestingly, these biomarkers may differ between PD and atypical parkinsonisms (MSA or PSP), as higher neuroinflammatory biomarker levels reflect a more aggressive neurodegenerative disorder [28].

Combinations of various biomarkers in diagnostic multipanels will provide a useful tool to increase diagnostic accuracy of parkinsonisms [26,27], but further studies are needed to validate these findings in clinical practice. Biomarkers may be useful to characterize not only different pathologies, but also different disease subtypes, which have different biological substrates and prognosis [30]. In this field, biomarkers will provide a biological basis to stratify patients in clinical trials of disease-modifying drugs and to personalize treatment according to a specific biological profile [31].

### 2.2. Disorders of Consciousness

Acute Disorder of Consciousness (DoC), also defined as coma, can be a consequence of severe brain injury or systemic disorders (from cerebrovascular diseases, infectious diseases, brain hypoxia and more) [32].

Some comatose patients evolve towards a chronic DoC, being vegetative state [also known as unresponsive wakefulness syndrome (UWS)] [33] and minimally conscious state (MCS) [34]. Such states, characterized by impaired awareness and responsiveness, can be distinguished with a behavioral assessment through validated clinical scales such as the Coma Recovery Scale revised [35], although misdiagnosis is possible. However, as clinical assessment is based on research of motor non-reflex responses to certain stimuli or orders, these scales have important limitations. Using behavioral evaluation only, signs of awareness can be missed. In fact, functional neuroimaging studies showed activity in specific brain areas of patients with chronic DoC in response to motor orders, hinting signs of consciousness.

The correct diagnosis is important as prognosis and functional outcome of patients with DoC seem to be better than in those with UWS [36]. It must also be considered the cause of brain damage, patient’s age, and the presence of comorbidities (Table 2) [37]. The Multi-Society Task Force on Persistent Vegetative State established a very low rate of awareness recovery with a 3 months cut-off on patients with hypoxic damage and a 1-year cut-off in patients with traumatic brain injury, if patients did not show signs of recovery before such time limits [38]. Most recent studies have fortunately showed recovery over a wider time range [39].

The main treatment for patients with DoC is neurorehabilitation, which should be started as soon as clinical stability has been reached. Admission of DoC patients to specialized neurorehabilitation units seems to improve functional outcome [40]. In DoC patients, rehabilitative goals are improvement of awareness and of voluntary motor function, and several prognostic factors have been described. Most importantly, it has been shown that prognosis worsens over time, although signs of recovery have also been observed in chronic patients [41].

Several approaches have been tried to enhance awareness and responsivity. Pharmacological interventions and both invasive and non-invasive brain stimulation have been proposed as DoC treatments, together with conventional neurorehabilitation, aimed to stimulate brain plasticity [42]. However, few treatments have shown some efficacy in the recovery of consciousness, such as amantadine and transcranial direct current stimulation (tDCS) [43,44]. Recovery of consciousness is still a large challenge for clinicians; among neurorehabilitation strategies, sensory stimulation with personalized target stimuli has been used to attempt and increase awareness in DoC patients [45].

## 3. Cardiovascular Diseases: Focus on the Acute Coronary Syndrome

Cardiovascular diseases (CVD) represent multifaceted conditions in which individual predisposition and environmental factors intersect to generate the disease, and omic techniques might help to understand the biological processes underlying them, by analyzing the interactions between heterogeneous components [46]. The biological complexity of diseases could be explored at different levels. Given a disease such as acute coronary syndrome (ACS), these levels go from the clinical approach, aimed at identifying the clinical symptoms and signs, to the basic science and pharmacological approach, based on the identification of disease specific molecular pathways and biomarkers, and on the molecular effects of different drugs [47].

As far as ACS are concerned, over the last century we have learned that, despite the same clinical presentation (e.g., chest pain and electrocardiogram [ECG] alterations), different pathophysiological mechanisms could underlie the disease. Postmortem studies carried out in the 1980s proposed that plaque rupture, characterized by a large central lipid core, abundance of inflammatory cells and a thin fibrous cap, caused most fatal myocardial infarctions. However, subsequent pathological studies showed that most thin-capped fibro-atheroma are clinically stable, with <5% of them causing clinical events over 3 years of follow-up. Moreover, inflammation may not drive all transitions from stable atherosclerosis to acute thrombotic events, as demonstrated in studies showing that about half of ACS occurred in the presence of normal levels of C-reactive protein (CRP), a marker of inflammation [48]. Finally, about one fifth of ACS occur in the apparent absence of coronary thrombosis, suggesting that functional alterations of the arterial wall can contribute to ACS pathogenesis beyond thrombus formation.

To date, there are at least four different mechanisms identified as possible pathological pathways to ACS: plaque rupture with systemic inflammation, plaque rupture without systemic inflammation, plaque erosion, and plaque without thrombus (Table 3) [49].

### 3.1. Plaque Rupture with Systemic Inflammation

Increased levels of the inflammatory biomarker CRP represents the main clue of the involvement of systemic inflammation in ACS [49]. Activated macrophages that abundantly infiltrate the region of the ruptured fibrous cap in about two thirds of patients with ACS are actively involved in the elaboration of enzymes that degrade arterial extracellular matrix, such as matrix metalloproteinases [69]. An altered adaptive immunity is also strongly associated with the pro-inflammatory profile of this kind of ACS [70]. Patients with ACS have an increased population of particularly pro-inflammatory CD4+ lymphocytes characterized by low cell surface expression of the costimulatory molecule CD28, together with a reduced number and suppressive function of circulating regulatory T cells (Tregs) compared with patients with stable angina and healthy control subjects [71,72,73]. The molecular mechanisms responsible for the T-cell imbalance and inflammation in ACS remain largely unknown and the infections hypothesis might only partially explain the onset of the inflammatory triggers leading to plaque progression and disruption [51]. Moreover, the regulatory mediators upstream T-cell receptor plays a pivotal role in the differentiation and modulation of T-cell number and functions, such as CD31 and protein tyrosine phosphatase N22 [73,74].

### 3.2. Plaque Rupture without Systemic Inflammation

Several other mechanisms others than inflammation may contribute to plaque rupture, including extreme emotional disturbance (i.e., earthquakes or a beloved loss), or acute manifestations of long-term emotional disturbances (i.e., chronic diseases), intense physical exertion and local mechanical stress at the level of the artery wall [75]. The precise causes of instability remain poorly understood, since this kind of plaque instability has undergone less extensive investigation so far. The relationship between psychological stress and plaque rupture may suggest a prominent role of the increased catecholamine release in triggering instability for those plaques already prone to rupture due to local alteration of plaque composition [76]. Cholesterol crystal formation in the lipid core is associated with higher risk of plaque rupture and thrombosis, together with inflammasome activation, and increased interleukin (IL)-1 and IL-18 production [56].

Of note, even with different triggers and effectors, when compared with plaque instability induced by systemic inflammation, a subclinical pro-inflammatory microenvironment around the plaque might also contribute to events leading to coronary instability [77].

### 3.3. Plaque Erosion

Superficial erosion has a distinct epidemiology, being more prevalent in women than men, and is characterized by a different plaque morphology and different pathophysiological mechanisms [60,61]. Neutrophil activation seems to play a pivotal role in thrombosis due to plaque erosion, with higher systemic myeloperoxidase levels if compared to patients with plaque rupture [78]. Eroded lesions contain few macrophages or T lymphocytes and abundant quote of proteoglycans, glycosaminoglycans and arterial smooth muscle cells (SMCs). The loss of intimal endothelial cells layer, together with thrombus formation, histologically defines eroded lesions. On the other hand, the optical coherence tomography (OCT) diagnosis of plaque erosion remains a diagnosis by exclusion: clinical signs and symptoms of ACS and OCT evidence of mural thrombus without clear signs of plaque fissure [52]. Recent works have demonstrated the involvement of an altered hyaluronan (HA) metabolism with increased expression of the HA receptor CD44 and of the degradation enzyme hyaluronidase-2 (HYAL2) in promoting the susceptibility of endothelial cells to apoptotic stimuli [53].

### 3.4. Plaque without Thrombus

In patients with ACS without plaque thrombus, the cause of acute ischemia seems to rely on a functional alteration of coronary circulation involving large epicardial coronary arteries or the coronary microcirculation. Intracoronary acetylcholine administration elicited coronary spasm in nearly 50% of patients with suspected ACS and no culprit lesions at coronary angiography [54]. Of note, a positive provocative test for spasm identifies a high-risk subset of patients [55]. Coronary artery spasm and endothelial dysfunction may be also present in patients with obstructive atherosclerosis and have been associated with coronary instability and/or persistence of symptoms after percutaneous coronary intervention (PCI) [79]. Myocardial ischemia has been also associated with microvascular spasm [56]. This mechanism represents the major determinant of Takotsubo cardiomyopathy, which frequently occurs in the absence of obstructive atherosclerosis [80]. The pathophysiological mechanisms underpinning both epicardial and microvascular spasm may result from impaired vasodilatation or from vasoconstrictor stimuli acting on hyperreactive vascular SMCs [61]. Incidentally, an increase in Rho-kinase activity seems to be the major mechanism of SMC hyperreactivity [57].

The large body of knowledge around plaque rupture has led to an increased control of traditional risk factors such as hypercholesterolemia, hypertension, and smoking. This has consequently induced a gradual shift towards other mechanisms leading to the clinical presentation of ACS. For this reason, we are increasingly in need for a tailored therapeutic approach to apply also to the prevention and treatment of the less well-recognized pathways to acute myocardial ischemia. We should systematically link our clinical approach to CVD to their pathophysiological mechanisms, by discovering and developing molecular and imaging biomarkers reflecting the underpinning mechanism that yields acute ischemia in ACS. This approach may help in understanding the multiple underlying causes of ACS for a better diagnosis and a precise deployment of treatment strategies [52].

In this scenario, multiomic technologies might be a viable instrument to unravel the intricate molecular networks standing behind the clinical presentation and possibly resulting from different pathophysiological pathways [58]. While using this powerful tool, every investigator must understand the limitations arising from the interpretation of very large, complex and non-linear datasets and should confirm the findings with thoughtful and well-designed experiment to link molecular networks to pathophysiological mechanisms [47].

## 4. The Example of Inflammatory Bowel Diseases

Inflammatory Bowel Diseases (IBD) represent a paradigmatic example of chronic disorders with complex etiopathogenesis. Clinically-based nosographic classification of IBD divides them into two main clinical phenotypes (Crohn’s disease [CD] and ulcerative colitis [UC]), and for each phenotype a classification based on endoscopic and radiological findings (i.e., Montreal classification) is adopted [81,82]. However, such an approach is associated with significant limitations.

First, it fails to adequately express the actual clinical heterogeneity of IBD, nor has it a meaningful impact on patients’ prognosis; furthermore, there is increasing evidence that even the same clinical sub-phenotype might rely on different pathogenic pathways in different patients [83,84]. The exact etiopathogenesis of IBD has not been completely elucidated, but it is now commonly considered that they are multifactorial disorders that arise from the complex interactions of environmental triggers with immunological and microbial features in a genetically susceptible host (e.g., Table 4) [85,86].

Indeed, the hypothesis had been proposed that IBD actually represent a family of similar but distinct disorders, each of which is characterized by specific pathogenic disfunctions [86]. This hypothesis finds confirmation in data from Randomized Clinical Trials (RCTs), which point out that roughly half of the patients respond to a certain medical therapy [90] despite sharing somewhat homogenous clinical characteristics; such observations seemingly suggest that the pathogenic variability of IBD might be even greater than the already notable clinical heterogeneity.

Furthermore, it has been demonstrated that the immunological landscape of a patient with IBD is rather dynamic and evolves over time: for instance, in CD postoperative recurrence, a progressive transition has been observed from a prevalent Th1 phenotype (characterizing the phases preceding recurrence detectable through endoscopy) towards a mixed Th1/Th17 phenotype when endoscopically detectable established lesions occurred [87]. Certain cytokines can play pleiotropic and often dichotomous roles, depending on the immunological environment or the presence of other non-immunological stimuli. Interleukin (IL)-22 and IL-33 represent a paradigmatic example of double-edged sword cytokines in intestinal inflammation: depending on the “phase” of inflammation, they can exert a pro-inflammatory role or promote epithelial regeneration and the closure of intestinal ulcers [88,89].

In the last decade, constantly growing attention has been paid to human gut microbiota. Far from being an inert and isolated bystander, it has emerged with a predominant role in a variety of human diseases (even not strictly related to the gastrointestinal tract), such as atherosclerosis [91], autism [92], diabetes [93], psoriasis [94], spondylarthritis [95], as well as IBD [85], obesity [96], irritable bowel syndrome [97] and various cancers [98]. The mutual influences between the immune system and gut microbiota and the inter-kingdom connections between gut microbiota and other organs have been proposed as major players in influencing the severity of certain diseases and in determining the efficacy of specific therapies [99].

It has been demonstrated that gut microbiota exerts an important regulatory role in IBD: it can influence disease progression and aggressivity, as well as response to specific therapies [85]. However, it is worth mentioning that the influence of gut microbiota extends beyond just gastrointestinal disorders. For instance, researchers found a link between the presence of specific bacterial species and responsiveness to immunotherapy in cancer: in a murine model of melanoma, oral administration of *Bifidobacteria* significantly enhances the efficacy of programmed cell death protein 1 ligand 1 (PD-L1)-specific antibody therapy (checkpoint blockade), via higher CD8(+) T cell priming and accumulation in the tumor microenvironment [100]. Another interesting line of evidence comes from a recent study on type 2 diabetes, where the authors observed that 2 species of *Lactobacilli* could improve lipid metabolism in mice fed with western diet, by upregulating oxidative phosphorylation pathways in hepatic mitochondria [99].

Different omes (mainly genome, exposome, immunome and microbiome) contribute to the etiopathogenesis of IBD. Such -omes have been traditionally investigated separately, without attributing the rightful attention to their interactions, which are indeed likely to play an important role in explaining the heterogeneity of inflammatory pathways that can sustain intestinal damage in IBD. Notably, researchers proposed a novel model of interpreting the different factors (called “interactome”). The model contributes to IBD pathogenesis, based on dissecting and discovering the networks existing between these multiple omes; this model is defined as “a disease network in which dysregulation of individual omes causes intestinal inflammation mediated by dysfunctional molecular modules controlling all biological responses” [101].

## 5. Results

Personalizing clinical phenotypes is a challenge for research and biomedical practice. Experiences in the field may help in understanding which techniques and tools are the best performing to support the development of potential clinical phenotype personalization. In 2018, the Italian Ministry of Health recognized our institution Fondazione Policlinico Gemelli in Rome as a research hospital for the disciplines of “Personalized Medicine” and “Innovative Biomedical Technologies”. In our research hospital, we conduct several studies to increase a deeper understanding on this topic. The connection between practical experiences and scientific literature may highlight the potential of personalizing clinical phenotyping towards multiomics, systems medicine, and Artificial Intelligence (AI).

### 5.1. Personalized Medicine for Parkinson’s Disease

Biomarkers found several applications in PD research: diagnosis, differentiation of disease subtypes, prognosis, disease monitoring, response monitoring to a specific drug and personalized treatment [23]. Levodopa-induced dyskinesias (LIDs) are a typical complication of long-term dopamine repletion treatment in PD, and they highly affect the quality of life [102]. Pathophysiology and genetics of LIDs are fields of great interest and recent development in PD research, and future discoveries expanding our knowledge in these fields might lead to prevention and treatment strategies for LIDs [103], thus improving the quality of life of people with PD.

In this context, a project about biomarkers in PD is ongoing at our Neurology Unit. This is a cross-sectional multicenter study, which has the following objectives:

1. to characterize clinical and genetic features and measure the CSF biomarkers in dyskinetic and non-dyskinetic PD patients, in order to test in vitro the modulation of molecular and neuronal LIDs correlates;
2. to identify the role of pre- and post-synaptic molecular targets associated to the accumulation of α-synuclein that may contribute to the synaptic alterations underlying LIDs;
3. to dissect the role of *LRRK2* biology and phosphorylation in the signaling cascade downstream D1 receptors and glutamate receptor activation associated to the synaptic alterations underlying LIDs;
4. to identify compounds able to induce *LRRK2* phosphorylation or to reduce α-synuclein aggregation and verify the effects of these molecules in PD animals in the prevention of dyskinetic motor behavior following levodopa treatment.

One-hundred PD patients with idiopathic PD on treatment with levodopa will be included in the study. Subjects with cognitive impairment, on treatment with anticoagulants or antiplatelet drugs and pregnant women will be excluded. Enrolled patients undergo clinical evaluation with validated scales for PD motor and non-motor symptoms [Unified Parkinson’s Disease Rating Scale—Movement Disorder Society (MDS-UPDRS)] [104], motor complications [Wearing-off Questionnaire (WOQ-19) and Unified Dyskinesia Rating Scale (UDyRS) [105,106] and cognitive impairment [Montreal cognitive assessment (MoCA) [107]. Then, blood and CSF samples are collected.

The following analyses are performed on blood samples: glucocerebrosidase (GBA) gene sequencing and research of common mutations of the *LRKK2* gene (p.G2019S, p.R1441C/G/H, p.N1437H, p.Y1699C, p.I2020T). Mutations in the *GBA* gene are numerically the most important risk factor for developing Parkinson disease (PD) accounting for at least 5% of all PD cases [108], while mutations in the *LRKK2* gene were found in 0.5% to 2.0% of sporadic PD and 5% of dominantly inherited familial parkinsonism [109]. In CSF samples, the following biomarkers are analyzed: total and oligomeric α-synuclein, Aβ-40, Aβ-42, total tau, phosphorylated-tau, neurofilament light chain (Nfl), neurogranin, *LRRK2* (with the analysis of phosphorylation at sites Ser 910, 935 and 1292), DJ-1, YKL-40, enzymatic activity of β-glucocerebrosidase, cathepsin D and prosaposin.

These biomarkers, reflecting several pathophysiological mechanisms of PD (accumulation of misfolded proteins, impairment of autophagy-lysosomal system, mitochondrial dysfunction, neuroinflammation and neurodegeneration), will be analyzed to biologically characterize dyskinetic and non-dyskinetic PD patients. Moreover, the use of AI and Machine Learning (ML) algorithms will help define discriminative models using biomarkers [110], integrating them in clinical settings to assist more accurate and informed decision making.

Finally, in this project, adeno-associated virus (AAV) α-synuclein and *LRKK2* knockout rat models of PD will elucidate the pathogenic role of α-synuclein aggregation and LRKK2 in LIDs, as well as their interaction, providing further pathophysiological evidences that will be translated and integrated with clinical data, allowing personalized prevention and treatment strategies for LIDs in PD.

### 5.2. Personalized Medicine for Disorders of Consciousness

The efficacy of most neurorehabilitative interventions have a common bias related to the difficulty in distinguishing improvements caused by the intervention and those because of spontaneous recovery and behavioral compensation and strategies, in particular in acute care settings [45]. Despite patients having similar clinical manifestations, the therapeutic outcome may differ among them. DoC treatment vary depending on etiology, intensity, and stages of the condition. Due to the nature of injury, rehabilitation does not lend itself to a singular “protocolized” plan of therapy. Yet, by nature and by necessity, rehabilitation medicine operates as a functional model of personalized care. Therefore, personalization of the treatment is crucial for achieving the best clinical outcome.

Sensory stimulation is a rehabilitative intervention aimed at improving arousal and responsiveness in patients with DoC [45]. Signs of functional improvement have been described in animal models with severe brain injury [111] and studies show that a complex stimulating environment can stimulate plasticity and potentially induce brain recovery in patients with severe brain injury [112]. Many studies have focused on the impact on sensory stimulation in the rehabilitation of DoC patients, and recent revisions have established how little evidence of efficacy exists. Studies were very heterogeneous, with different patients’ selection and stimulation protocols (with different stimulation modes, times, duration). Similarly, outcome measures vary from behavioral assessment to detection of spontaneous movements, to autonomic nervous system (ANS) responses measurements [45].

A review by Padilla provided strong evidence for the effectiveness of multisensory stimulation programs to improve arousal [113]. Autobiographical and emotional stimuli relevant for patients are more effective than generic stimuli: contents will be likely to impact the patient more or less on the basis of the patient history and life experience [45]. Recently, several studies considered biographical background, such as socio-cultural baggage, hobbies, etc. of patients to better address rehabilitation treatment in neurodegenerative diseases [114].

Based on such preliminary evidences, our High Intensity Neurorehabilitation Unit is carrying out a project in order to assess the effects on arousal and responsiveness of DoC patients of personalized emotionally salient sensory stimulation delivered with multimodal fashion (auditory-visual) in an immersive non-interactive setting: the Multisensory Cave.

Such an immersive virtual reality setting creates a digitally simulated experience with multimodal stimuli to create the sensation of being in the real world. This setting consists of a large room where high definition videos are projected on a three-wall screen (front wall and side walls) surrounding the patients over 270° and the audio is provided by two high definition speakers and a subwoofer. The audiovisual content aims at providing a reconnection to the world through familiar images and sounds. Personalized emotionally salient stimuli are chosen after a meeting with family members and individually customized.

Patients with DoC may access to the High Intensity Neurorehabilitation Unit by different emergency departments at our research hospital; each patient undergoes a 20 min daily stimulation session for one month. In the first two weeks, general stimuli are used, the same for all patients. In the second two weeks, personalized stimuli are delivered. During the session, multiple parameters are measured: vital signs, behavioral responses, and ANS activity. Behavioral responses, recorded by a skilled neuropsychologist include eye opening, responsiveness to sensorial stimuli (verbal/tactile/nociceptive), spontaneous movements, changes of contact with the environment (such as visual exploration, vocalizations), and agitation.

These data were recorded as dichotomous variable (presence/absence). ANS function is explored by measurement of electro dermal activity (EDA) with specific sensors. Diagnostic behavioral scales (Coma Recovery Scale revised) and high-density EEG is recorded before the beginning of the first session, at two weeks cut off and at the end of the stimulation protocol. We hope that a personalized stimulation approach will prove useful in promoting arousal and recovery of responsivity in DoC patients.

There is limited research focusing on predictive biomarkers and functional outcomes for this health condition. To better address the most appropriate rehabilitation treatment on patients’ needs and life experience, we can consider the impact of omics [115]. Thus, the challenge of neurorehabilitation is to identify a viable way that provides a Systems Medicine approach to examine multiparametric data and multidimensional outcomes (e.g., neurobiology of injury response to treatment). Considering the effectiveness of the sensory stimulation—especially using emotional pathways known to the patient, it would be interesting observe neurophysiological changes (before/during/after a personalized stimulation) by High Density Electroencephalography (HD-EEG), and how brain is working by using Positron Emission Tomography (PET), to assess the “personalization” of the treatment in terms of type, quantity and duration.

Research suggest AI and ML algorithms can drive towards more effective treatment by data mining of clinical and paraclinical signs (Glasgow Coma Scale Score, base deficit, and diastolic blood pressure) of injured patients after traumatic injury resuscitation [116].

### 5.3. Personalized Medicine for Acute Coronary Syndrome and other Cardiovascular Diseases

In our Institution, the Cardiovascular Unit contributed in the abovementioned differentiation of ACS pathogenic pathways concerning plaques. However, omics experiments in cardiology are catalyzing increasing attention [117]. Regarding genomics, traditional genome-wide association studies (GWAS) are sequencing approaches used to associate specific genetic variations with particular diseases and have been undertaken also for CVD, including heart failure, ACS, atherosclerosis. Genomic studies identified the association of several single-nucleotide polymorphisms (SNPs) with CVD and their interactions with common cardiovascular risk factors. In a recent study, 1.7 million SNPs were evaluated for linkage with coronary artery disease (CAD) and used to generate a genomic risk score. Based on an individual’s genetic profile, this score provided an estimated risk of incident CAD, and remained predictive after corrections for common environmental and clinical risk factors [62].

Indeed, the major issue of genomic analysis is that the discovered linkages are often far to be explained in terms of mechanisms and the powerful effects of behavioral and environmental factors, such as smoking, diet and socioeconomic status are not included in the information that can be drawn from genomic analyses. This limitation has been partially overcome through Mendelian randomization studies, examining the effects of modifiable exposures on the functions of a known gene and thus on the derived disease [63]. For example, our Cardiovascular Unit found that, despite the fact that the plasma levels of C-reactive protein (CRP) are considered a biomarker in CAD and are related to outcome and prognosis, a Mendelian randomization study determined that these levels were not causal and, therefore, CRP has not be considered a potential drug target for the treatment of CAD [50,59].

Epigenomic and transcriptomic represent a step forward to evaluate the interactions between genes in their expression, rather than comparing genome and phenotype. The quantification of mRNA in a tissue or in a specific cell subtype provide information about the differential gene expression and the interaction between different genes [64]. This allows understanding if specific loci might be associated to increased individual risk of CVD, and if a network of interactions among different genes might potentiate this risk. To increase the complexity of these networks, CVD risk is often mediated by different cell types, in which the same gene expression might play different function. miR-126 is a miRNA abundantly expressed in endothelial cells, where it plays a pivotal function for endothelial integrity [65]. Alongside, platelet reactivity is positively correlated with its levels in megakaryocytes [64]. However, miR-126 related genotype has not been associated to increased CVD in any GWAS analysis so far. This example demonstrates how gene networks existing at different levels might alter the risk profile.

Proteomics technologies allow the discovery and quantification of proteins in a biological sample and make it possible to also evaluate the whole set of molecular interaction in cells, samples, and tissues, which are crucial to understand biological mechanisms. Integrating GWAS studies with data on protein-protein interactions has helped to better identify putative susceptibility genes related to CVD [66].

Metabolomic and lipidomic studies aim at identifying small molecules involved in CVD; a metabolomic platform has been recently used to analyze plasma from the participant of the PROSPER study of pravastatin and has demonstrated that the metabolomic effects of a loss-of-function PCSK9 variant and those of therapy with statins were similar when assessing the lipidic profile [67]. These findings further proved that lowering plasma LDL levels reduces the risk of CVD, and, by demonstrating that the changes in plasma LDL-cholesterol levels are similar with statin and PCSK9-inhibitor therapies, confirm the clinical success of the PCSK9 inhibitor class of drugs [68].

ACS prediction remains a challenge for clinical practice. AI and ML, associated with trained emergency personnel, could help improving clinical outcomes in patients with chest pain [118] and predicting needs for urgent revascularization in emergency patients [119].

### 5.4. Personalized Medicine for Inflammatory Bowel Diseases

Regarding IBD, it has been shown that small populations of rare cells can exert crucial regulatory roles in immune-mediated diseases [120]. Single-cell analysis has proven an exceptionally useful tool for the identification and functional characterization of these rare cells. For instance, this type of analysis could offer a deeper understanding of IBD pathogenesis and help to identify new therapeutic targets; our Internal Medicine and Gastroenterology Unit with the Surgical Endoscopy Unit are currently performing single-cell transcriptomic studies in multi-refractory IBD patients receiving combinations of targeted therapies for uncontrolled intestinal inflammation [121]. This approach could offer the opportunity to—at least partially—elucidate the mechanisms behind treatment refractoriness in IBD and, hopefully, give some hints on the efficacy of contemporarily blocking multiple inflammatory pathways. For the same reason, we are currently developing in vitro cellular and tissue models to study the potential synergistic effects of multiple drugs on inflammatory cells.

However, the emergent properties of biological systems cannot be overlooked—their functions cannot be solely understood by studying each component, as some functions are the result of the interactions among different components. Data from multiomic analysis need to be integrated in multiparametric models. For instance, the advanced concept of IBD interactome [83] and a growing attention in translational and clinical research can elucidate how networks underlying diseases’ pathogenesis work.

The potential of this integrated approach can be intuited if we look at some recent works where candidate predictive models for response to therapy have been developed: multiparametric models (incorporating clinical, genetic, immunological and microbiological features) have performed quite well in predicting a patient’s response to therapy, proving to be superior to single predictive factors [122].

We are currently exploring a similar approach in collaboration with rheumatologists (given that IBD often overlap with some forms of arthritis, mainly seronegative spondylarthritis), to create a predictive model of response to biological therapies based on the integration of clinical, microbiological and immunological features.

Microbiota manipulation via Faecal Microbiota Transplantation (FMT) has been proposed for the treatment of many gastrointestinal disorders [123]. Interesting outcomes have been obtained in recurrent *Clostridium difficile* colitis [124], while more modest results have been observed in IBD, the most notable ones being in mild-to-moderately active UC [125]. This might probably be attributable to the fact that donor selection is less important in *Clostridium difficile* colitis, as it is caused by the uncontrolled overgrowth of a single pathobiont that can be tackled by simply replacing a dysbiotic microbial flora. When it comes to more complex diseases, it is likely that an accurate donor selection will play a more important role [126], as the transferred microbiota has to struggle against more intricate alterations sustained by a chronic trigger (such as the constant over activation of inflammatory response in IBD, for instance). Extensive characterizations of gut microbiota composition and functions, as well as their interplay with the immune system, have been a major focus of study for our group [127]. The biunivocal interconnections between gut microbiota and mucosal immunity are yet to be completely elucidated, but they are likely to play a pivotal role in determining how the human system reacts to environmental triggers and, therefore, in the initiation of non-homeostatic, pathological responses.

Finally, it is worth mentioning the expanding role of gastrointestinal endoscopy in PM. For instance, in IBD, with the advent of AI and ML, there has been hope that these new technological tools might be able to overcome the poor predictive value and the interobserver variability that characterize tradition endoscopic scoring systems. A notable example is represented by the recent work from Takenada et al, who developed and validated a deep neural network for the assessment of UC that could predict endoscopic and histologic remission with 90.1% and 92.9% accuracy, respectively [128].

## 6. Discussion and Conclusions

Advances in PM are eagerly required for two main reasons: to reduce health-related expenditure (which has been constantly rising in western countries in the last decades and may not be affordable anymore on the long-term) and to improve the quality of assistance providing most appropriate treatments, for each patient, at the right time. Considering the heterogeneity of clinical features and pathogenic pathways that can trigger certain diseases, it comes as overall unsurprising that a solely genomic understanding can be of limited efficacy to differentiate a diagnosis or personalize a treatment. Such a high level of complexity requires an approach based on the precepts of Systems Medicine. As we acquire more and deeper data from different types of analysis (genomic, proteomic, transcriptomic, immunophenotyping, microbiological, etc.), the complexity that lies behind certain clinical phenotypes is progressively unveiled. However, our comprehension of the exact mechanisms behind the complexity of certain disorders is still initial. As human systems are non-linear systems [129], where small alterations can produce significant macroscopic effect over time, a more granular approach is needed (multiomics); meanwhile, it is also fundamental to analyze the overall complexity of each person if we are willing to tailor a diagnosis or a treatment through Systems Medicine.

Since analyzing a significant volume of heterogeneous data from several sources is not effortless, computational models can support clinicians in their daily work for early diagnosis and prognostic support (e.g., simulation of a response to treatments) towards the AI/ML calibration on different clinical and non-clinical features. The application of this approach could reduce time-consuming tasks during the diagnostic, therapeutic, and prognostic workflow. Future research and clinical experiences may elucidate further strategies for developing a Systems Medicine approach towards the integration of different omics and AI/ML solutions to identify the best predictors for patients’ stratification, to personalize the identification of disease pathways, as well as to predict diagnostic and potential deep phenotyping complex diseases [130].

## Figures and Tables

**Table 1:** Degenerative Parkinsonisms: diseases and principal biomarkers.

Disease	Biomarker	Reference
Parkinson’s disease (PD) vs. atypical parkinsonisms [Multiple System Atrophy (MSA), Dementia with Lewy bodies (DLB), Progressive Supranuclear Palsy (PSP), Cortico-basal degeneration (CBD)] MSA vs. DLB	Accumulation of proteinaceous material (different dosage): α-synuclein, Aβ-40/Aβ-42, phosphorylated tau (p-tau)/total tau	[24,25,26,27]
Parkinson’s disease (PD) vs. atypical parkinsonisms (MSA, PSP, CBD)	Neurofilament light chain (Nfl)	[26]
Parkinson’s disease (PD) vs. atypical parkinsonism (MSA, PSP, CBD)	Neuroinflammation (acute phase proteins, cytokines and markers of microglial activation)	[26,28]

**Table 2:** Disorders of Consciousness: diseases and principal biomarkers.

Disease	Biomarker	Reference
Disorders of Consciousness (all phenotypes)	• Standard: Coma Recovery Scale revised	[35]
Systemic disorder	• Vegetative state (awareness, consciousness, responsiveness to motor orders) • Age • Presence of comorbidities	[33,34] [37] [37]
Traumatic disorder	• Cause of the brain damage • Vegetative state (awareness, consciousness, responsiveness to motor orders) • Age • Presence of comorbidities	[32] [33,34] [37] [37]

**Table 3:** Acute Coronary Syndrome: diseases and principal biomarkers.

Disease	Biomarker	References
Acute Coronary Syndrome [ACS] (all phenotypes)	• Levels of C-reactive protein (CRP) • Plasma Low Density Lipoprotein (LDL)-cholesterol levels • Loss-of-function Proprotein convertase subtilisin/kexin type (PCSK9) variant	[49,50] [46,47,51] [47,48]
ACS (plaque rupture with systemic inflammation)	• Pro-inflammatory CD4+ lymphocytes with low cell surface expression of the costimulatory molecule CD28 • Reduced number and suppressive function of circulating regulatory T cells (Tregs) • Role of the regulatory mediators upstream of the T-cell receptor in the differentiation and modulation of T-cell number and functions, such as CD31 and protein tyrosine phosphatase N22	[52,53] [54,55] [54,55]
ACS (plaque rupture without systemic inflammation)	• Inflammasome activation • Interleukin (IL)-1 • Interleukin (IL)-18 • Catecholamine release due to emotional disturbance;	[56] [56] [56] [49]
ACS (plaque erosion)	• Neutrophil activation • Macrophages or T lymphocytes • Proteoglycans • Glycosaminoglycans • Arterial Smooth Muscle Cells (SMCs) • Increased enzyme hyaluronidase-2 (HYAL2) expression of monocytes • Increased CD44 expression of endothelial cells	[57,58] [59] [52,60,61] [52,60,61] [52,60] [53,59,62,63] [63]
ACS (plaque without thrombus)	• Microvascular spasm • Rho-kinase activity	[64,65,66,67] [68]

**Table 4:** Inflammatory Bowel Diseases: diseases and principal biomarkers.

Disease	Biomarker	Reference
Crohn’s Disease (CD) Ulcerative Colitis (UC)	• Genetic polymorphisms (NOD2, genes of TNF pathway, apoptosis-related genes) • C-reactive protein levels • Faecal calprotectin levels • Autoantibodies (ANCA, ASCA) • Drug trough levels • Anti-drug antibodies • Microbiota diversity and composition	[84,85,86]
CD postoperative recurrence	• Progressive transition from Th1 to Th1/Th17 immunophenotype	[87]
Colonic inflammation (UC, possibly colonic CD)	• Interleukin (IL)-22 and IL-33 as dichotomous cytokines (can either promote intestinal inflammation and wound repair)	[88,89]
TNF-resistance	• Oncostatin M • TREM-1 • Reduced gut microbiota metabolic interchanges	[83,84]

## Data Availability

Not applicable.
